# Pediatric kidney transplantation: is it safe to perform during night-time or day-off?

**DOI:** 10.1007/s00383-024-05666-4

**Published:** 2024-03-19

**Authors:** Filippo Ghidini, Marina Andreetta, Federica De Corti, Elisa Benetti, Enrico Vidal, Piergiorgio Gamba, Francesco Fascetti Leon

**Affiliations:** 1 Department of Women’s and Children’s Health, University of Padua, Padua, Italy; 2 Department of Pediatric Surgery, Hôpitaux Civils de Colmar, Colmar, France; 3 Department of Pediatric Surgery and Pediatric Minimally Invasive Surgery and New Technologies, San Bortolo Hospital, Vicenza, Italy; 4 Pediatric Surgery Unit, Department of Women’s and Children’s Health, University of Padua, Padua, Italy; 5 Pediatric Nephrology Unit, Department of Women’s and Children’s Health, University of Padua, Padua, Italy; 6 Department of Medicine, University of Udine, Udine, Italy

**Keywords:** Kidney transplantation, Children, Night-time, Complications, Fatigue, Sleep deprivation

## Abstract

**Purpose:**

To investigate the impact of after-hours surgery on the outcomes of pediatric kidney transplantation (KT).

**Methods:**

Medical records of pediatric KTs performed at a single institution between 2013 and 2021 were retrospectively reviewed. The population was split into three groups according to the incision time and calendar: ordinary day (8.00 AM – 6.30 PM), day-off, and night-time (6.30 PM – 8.00 AM). The following endpoints were compared: ischemia times, length of surgery, complications, delayed graft function (DGF), primary graft non-function (PGNF), and eGFR at three-month follow-up.

**Results:**

Ninety-six non-living donor KTs were performed, median age 11 (IQR 4.3–14) years and median body weight 26 (IQR 13–50) kg. Forty-one (43%) were performed during night-time and 28 (29%) during day-off. Ischemia times were similar (*p* = 0.769, *p* = 0.536). Day-off KTs presented an extended length of surgery (*p* = 0.011). Thirty-two complications were reported in 31 KTs. No difference in the overall rate of complications, DGF, PNGF, and three-month eGFR was found (*p* = 0.669, *p* = 0.383, *p* = 0.949, *p* = 0.093). Post-operative bleedings were more common in days-off (*p* = 0.003).

**Conclusion:**

The number of pediatric KTs performed during after-hours was considerable. Even though similar outcomes were reported, more caution should be focused on the KTs performed in days-off to avoid severe complications.

## Introduction

Kidney transplantation (KT) has been recognized as the first-choice treatment of end-stage kidney disease (ESKD) among children [1]. Even though improvements in the immunosuppressive therapy and perioperative management have been introduced, surgical complications remain a threat for the surgical and clinical outcome success of KT [2].

Several risk factors have been considered, including the surgical approach, the need for anticoagulants and for ureteral stenting [3–5]. Recently, human aspects, such as night-time or day-off surgery, have been investigated in the adult population [6]. Indeed, non-living donor KT is an emergent intervention, and, for this reason, the surgery might be performed after-hours during night-time or holydays. It has been hypothesized that after-hours surgery might raise the risk of complications, especially following KTs. This might be due to several factors, including professionals’ fatigue and lower availability of human and structural resources during after-hours [7]. On the other hand, the postponing of a non-living donor KT might prolong the cold ischemia time (CIT). This parameter increases the risk of delayed graft function (DGF) and eventually impacts on the outcome of the allograft [8].

As far as we know, the rate of surgical complications after pediatric non-living donor KT during night-time or day-off has not been investigated. Pediatric non-living donor KT might be more demanding and challenging compared to adult KT, especially in children weighing less than 15 kg [9]. For this reason, we hypothesized that night-time pediatric KTs could present a higher rate of surgical complications as compared with a day-time operation.

Even though the effects of surgeon’s fatigue are difficult to assess in real-life [10], reports from urgent and elective abdominal surgery in the adult populations found higher rate of mortality or complications during or immediately after weekends [11, 12]. We, therefore, hypothesized that the fatigue of ordinary working days could also impact the outcomes of the pediatric KTs performed during days-off.

The primary aim of this study was to report the distribution throughout the day of the surgical intervention for KT. A secondary aim was to compare the rate of surgical complications and the short-term survival of the allografts between the non-living donor KTs performed during after-hours (night-time or day-off) and those performed during on-duty hours.

## Materials and methods

### Study design

This was a single-center, retrospective, and observational study. Our Institutional Review Board has been notified (Prot. N° 25,618). All the legal guardians gave their consent for the data collection. STROBE checklist was followed for the study drafting.

### Population

The setting of the study was the Department of Women’s and Children’s Health of the University-Hospital of Padua, Italy.

Clinical records of the pediatric KTs performed at the Department between January 2013 and December 2021 were reviewed. All the non-living donor KTs with at least three-month follow-up were included. Living-donor KTs were excluded since the intervention is not considered an emergent procedure, performed among scheduled in-office time. Patients older than 18 years old at the time of KT were excluded.

For the purpose of the study, the population was split into three groups according to the incision time. The time lapse between 8.00 AM and 6.30 PM during working days was considered day-time according to the current policy of the University-Hospital. On the other hand, the interval between 6.30 PM and 8.00 AM was considered night-time. According to the Italian national calendar and the current policy of the University-Hospital, the third group included the KTs performed during day-time on Saturdays, Sundays, and public holidays.

### Immunosuppressive therapy and surgical procedure

For the whole study period, our institutional protocol for induction therapy included: methylprednisolone (500 mg/m^2^/die) and two doses of basiliximab (10 mg or 20 mg in patients with a body weight lower or greater than 35 kg, respectively), just before the transplantation and on day four after surgery. Within the first 24 h after KT, the maintenance therapy was initiated and included Tacrolimus, at an initial dose of 0.3 mg/kg aiming to a therapeutic trough level of 10–12 ng/ml, mycophenolate mofetil, at an initial dose of 600 mg/m^2^/die aiming to a therapeutic trough level of 1.5–3.5 mg/l, and methylprednisolone at an initial dose of 500 mg/m^2^/die, to be progressively reduced in the following weeks.

The surgical intervention was performed by a senior surgeon certified by the regional and national program for solid organ transplantations. The senior surgeon was assisted by another senior surgeon and two residents. During the study period, at least three senior surgeons were available for pediatric KT program. The 24-h on-call shift for KT changed every week. At least one pediatric KT was performed every month.

The main surgeon and the assistant used personalized optical loupes with a 2.5–4 times magnification and microsurgical instruments appropriated for patient age and vascular structures dimension. All the grafts were implanted into the iliac fossa through an extraperitoneal access. The right side was the first choice. The renal vein and artery were sutured in an end-to-side fashion to the iliac vessels or to the vena cava and aorta, in case of patients weighting less than 15 kg or in case of significant mismatch between donor’s and recipient’s body size. The ureteral–vesical anastomoses were performed through an extra-vesical approach according to the Lich–Gregoire technique. A trans-anastomotic external stent was inserted up to the renal pelvis in all the patients to preserve the patency of the anastomosis and to monitor the split urinary output of the transplanted kidney, especially in case of residual diuresis from the native kidneys.

The perioperative infusion of 5–10 units/kg/hours of unfractionated heparin was indicated in case of altered pre-operative coagulative screening, patients aging less than five years, weight less than 15 kg, considerable size mismatch (body weight ratio between donor and recipient higher than 1:4), donor kidney allograft with multiple vessels, apparent intimal lesion of the allograft renal artery, altered allograft perfusion immediately after implantation, such as venous congestion.

### Variables and endpoints

The following clinical variables were extracted from clinical records: gender, age at surgery, congenital anomalies of the urinary tract, kidney replacement therapy before KT, recipient’s body weight, donor–recipient body weight ratio (BWR), vascular anatomic variant leading to complex bench surgery procedures, and the need of anticoagulant therapy. The following endpoints were compared between the three groups: CIT, warm ischemia time (WIT), the length of surgery, the need for inotropic drugs in the early post-operative, the length of hospital stay, serum creatinine and eGFR, calculated according to bedside Schwartz’s formula [13] at discharge and after three months since the KT. The rate of surgical complications graded more than II, according to Clavien–Dindo Classification [14], were compared. Bleedings, graft venous thrombosis, arterial stenosis, urinary obstructions, new onset of medical conditions were considered adverse events. The rate of surgical re-interventions in the first 30 post-operative days was also assessed and compared among the groups. The occurrence of DGF and primary graft non-function (PGNF) was compared between the two Groups [15, 16].

### Statistics

The statistical analysis was performed using IBM^®^ SPSS Inc. Version 26.0. Categorical variables were reported as number (%) and continuous variables were reported as median value and inter-quartile range (IQR). The variables and the endpoints of the two groups were compared through a univariate analysis. Pearson’s chi-squared tests were used for categorical variables and one-way ANOVA tests were used for continuous variables. *P*-value ≤ 0.05 was considered as statistically significant.

## Results

### Population

One hundred and thirty KTs were performed during the nine-year study period. Among those, 96 (74%) were non-living donors urgent KTs. The median age and body weight at surgery were 11 (IQR 4.3–14) years and 26 (13–50) kg, respectively. Forty-five patients (47%) were females and fifty-one (53%) were males. According to the inclusion criteria, only 27 KTs (28%) were performed during ordinary days. Most of them was performed during after-hours. Twenty-eight KTs (29%) were performed during days-off and the remaining 41 (43%) were during night-time.

The demographic and perioperative characteristics of the groups were compared, as reported in Table 1. The rate of administration of anticoagulant therapy seemed to be more frequent during night-time (*p* = 0.052). However, when considering the distribution of recipients’ body weight among the groups, there was no difference in the rate of administration of anticoagulant therapy among night-time and the other groups (*p* = 0.312).

**Table 1:** Demographic and perioperative characteristics of the study population

	Ordinary-day KT (*n* = 27)	Day-off KT (*n* = 28)	Night-time KT (*n* = 41)	*p* value
Age at KT (median, IQR)	10 (3.7–13) years	13 (5.6–16) years	8.7 (4.3–13) years	0.698
Female gender (*n*,%)	12 (44)	13 (46)	19 (46)	0.985
Body weight (median, IQR)	24 (12–36) kg	32 (14–49) kg	22 (14–36)	0.414
Congenital anomalies of the urinary tract (*n*, %)	11 (41%)	9 (32%)	11 (27%)	0.486
Kidney replacement therapy (*n*,%)	Hemodialysis 5 (19%)	Hemodialysis 10 (36%)	Hemodialysis 10 (24%)	0.359
	Peritoneal dialysis 13 (48%)	Peritoneal dialysis 9 (32%)	Peritoneal dialysis 19 (46%)	
	Both 3 (11%)	Both 7 (25%)	Both 7 (17%)	
	Pre-emptive 6 (22%)	Pre-emptive 2 (7.1%)	Pre-emptive 5 (12%)	
Body weight ratio (median, IQR)	1.9 (1.0–3.0)	1.7 (1.0–2.3)	2.1 (1.0–3.5)	0.597
Low-weight (< 15 kg) patients (*n*, %)	9 (33%)	8 (29%)	11 (27%)	0.844
Vascular anatomic variants (*n*, %)	18 (67%)	17 (28)	19 (46%)	0.217
Anticoagulant therapy (*n*, %)	17 (63%)	14 (50%)	32 (78%)	0.052

### Clinical endpoints

The clinical endpoints were compared among the groups, as reported in Table 2. No difference was found in CIT (*p* = 0.769), WIT (*p* = 0.526), in the need for inotropic drugs (*p* = 0.168) and in the length of hospital stay (*p* = 0.571). Nevertheless, the length of surgery was longer for the KTs performed in the days-off (*p* = 0.011). As to the serum creatinine and the eGFR, no difference was found at discharge (*p* = 0.432, *p* = 0.521) and at 3-month follow-up (*p* = 0.371, *p* = 0.093).

**Table 2:** Clinical endpoints compared among the groups

	Ordinary-day KT (*n* = 27)	Day-off KT (*n* = 28)	Night-time KT (*n* = 41)	*p-value*
Cold ischemia time (median, IQR)	12 (10–13) hours	10 (8.5–12) hours	12 (10–15) hours	0.769
Warm ischemia time (median, IQR)	60 (59–65) minutes	65 (60–71) minutes	62 (55–69) minutes	0.526
Operative time (median, IQR)	255 (232–283) minutes	285 (251–330) minutes	235 (203–293) minutes	0.011
Inotropic drugs (*n*, %)	8 (30%)	15 (54%)	15 (37%)	0.168
Length of hospital stay (median, IQR)	19 (15–22) days	17 (15–24)	19 (14–24) days	0.571
Serum creatinine at discharge (median, IQR)	55 (36–82) μmol/l	81 (44–106) umol/l	57 (37–91) umol/l	0.432
eGFR at discharge (median, IQR)	78 (66–96) ml/min/1.73 m^2^	67 (54–94) ml/min/1.73 m^2^	77 (60–109) ml/min/1.73 m^2^	0.521
Serum creatinine at 3-month follow-up (median, IQR)	55 (43–79) umol/l	73 (54–112) umol/l	54 (36–69) umol/l	0.371
eGFR at 3-month follow-up (median, IQR)	76 (68–85) ml/min/1.73 m^2^	67 (50–82) ml/min/1.73 m^2^	81 (62–100) ml/min/1.73 m^2^	0.093
3-month graft loss (*n*, %)	0 (0%)	2 (7.1%)	4 (9.8%)	0.469

### Complications and adverse events

**Fig. 1:**
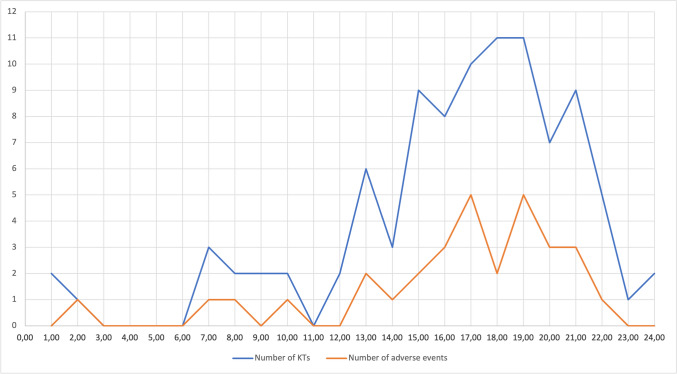
Distribution throughout the day of the cutting time for KTs and the percentage of adverse events

A total of 32 adverse events occurred in 31 patients. Figure 1 displays the distribution of the incision time throughout the day for the KTs affected by adverse events. The overall rate of complications was similar among the groups (*p* = 0.669), as reported in Table 3. However, the risk for post-operative bleeding was significant higher for KTs performed during days-off (0.003).

**Table 3:** Rate of complications and adverse events compared among the groups (*significant for *p* $\le$ 0.05)

	Ordinary-day KT (*n* = 27)	Day-off KT (*n* = 28)	Night-time KT (*n* = 41)	*p-value*
Overall complications *(n, %)*	6 (22%)	9 (32%)	10 (24%)	0.669
Bleeding, (n, %)	0 (0%)	6 (21%)	0 (0%)	0.003*
Graft venous thrombosis, (*n*, %)	0 (0%)	0 (0%)	3 (7.3%)	0.456
Arterial stenosis (*n*, %)	1 (3.7%)	0 (0%)	1 (2.4%)	0.412
Urinary obstruction (*n*, %)	2 (7.4%)	2 (7.1%)	4 (9.8%)	0.909
Medical conditions (*n*, %)	3 (11%)	6 (21%)	2 (4.9%)	0.458
Delayed graft function, (*n*, %)	0 (0%)	3 (11%)	3 (7.3%)	0.383
Primary graft non-function, (*n*, %)	0 (0%)	0 (0%)	1 (2.4%)	0.949

During the first 30 post-operative days, only one patient (3.7%) in the ordinary-day group required a further endourological procedure. Five patients (18%) in the day-off group required seven re-interventions, six laparotomies, and one endourological procedure. Finally, seven patients (17%) belonging to night-time group reported a further intervention for each of them. Four endourological procedures and three laparotomies. Even though the rate of re-intervention was higher in the after-hours KTs, no significant difference was found (0.211). The details about the re-interventions are described in Table 4.

**Table 4:** Re-interventions for adverse events during the first 30 post-operative days

	Ordinary-day KT (*n* = 27)	Day-off KT (*n* = 28)	Night-time KT (*n* = *41*)
Re-interventions	1 Ureteral stenting	2 Laparotomies for bleeding 1 Laparotomy for bleeding followed by graft removal for graft venous thrombosis 1 Graft removal for PGNF 1 Nephrostomy followed by re-do of the ureterovesical anastomoses	2 Nephrostomies 2 Ureteral stentings 2 Laparotomies for graft removal (1 PNGF, 1 graft venous thrombosis 1 Laparotomy for re-do of the arterial anastomoses

The occurrence of DGF and PGNF was similar (*p* = 0.383; *p* = 0.949).

During the three-month follow-up, no graft was lost among the KTs performed during working time. Two of the day-off group grafts (7.1%) failed because of venous graft thrombosis. In the night-time group, four graft failures (9.8%) were recorded: two cases due to venous graft thrombosis in patients weighting less than 10 kg, one case of PGNF, and one case for a recurrent glomerular disease after KT. No difference was found (*p* = 0.469).

## Discussion

In our series, less than 30% of non-living donor pediatric KTs were performed during the ordinary working hours. However, no differences in terms of short-term outcomes were found among the patients treated in the ordinary day, in the day-off or at night-time. The overall rate of complications was also similar. Nevertheless, KTs performed during days-off presented a longer length of surgery and a higher risk of post-operative bleedings.

Despite the institutional well-standardized protocol for pediatric KTs, the study presented some limitations that mainly resided in the retrospective design of the study. First of all, the limited size of the population might have undermined the statistical significance of the results. Second, it was not possible to assess the surgeon’s working and stress load before the KT surgical performance. This might represent a crucial aspect for the main objective of the paper. Future studies dealing with surgeon’s fatigue before pediatric KTs should be encouraged. Finally, the accurate duration of bench surgery was missing in most cases. However, the number of vascular variants requiring a complex bench surgery was similar. This aspect did not affect the outcomes or the risk of adverse events, as previously reported [17].

The identification of potential risk factors for complications is crucial to improve both performance and outcome of pediatric KTs. The outcomes of KTs performed not among regular working hours has been already investigated in the adult population. A systematic review and meta-analysis did not find an increased hazard for the KTs performed after-hours [18] and, more recently, two pilot studies found that night-time KT did not present an increased risk of complications [6, 7]. Nevertheless, pediatric KTs might be more challenging for the surgeons, especially those performed in low-weight children or in the presence of complex somatic and vascular malformations [3]. Furthermore, even though the weekend effect did not seem to impact on the outcomes of KTs in adult population [19], no data were available for children. For this reason, the study assessed the influence of the incision time and the calendar on the rate of adverse events, as already described for complex abdominal surgery in adult populations [11, 12].

Our rate of after-hours KTs was considerably high and might be explained by several aspects such as logistic. Indeed, the KT was frequently delayed after the ongoing elective surgery because no operating rooms were available. Moreover, KT was started immediately after a negative crossmatch testing was available, to reduce the length of the CIT, that represents one of the main factors influencing the recovery of the allograft [8]. For this reason, our current policy is to avoid delays for the performance of KTs.

More than two thirds of the KTs in our series were started between 5.00 and 9.00 PM. The surgeons might have just finished their ordinary activity without resting before entering on-call shift for KTs. This can certainly lead to sleep deprivation and fatigue [20]. Consequently, surgeon’s performance might be diminished, raising the risk of complications. It has been proved that sleep deprivation, fatigue, and stress due to the workload increase the risk of human error and prolong the surgical times for procedures that require concentration and caution [21]. Nevertheless, night-time KTs presented a reduced operative time, since the main operator could be more fatigued, willing to end earlier the intervention. On the other hand, KTs performed during days-off presented a longer length of surgery probably due to the fatigue accumulated during the previous ordinary working days.

Furthermore, most of the documented complications were concentrated in the span of time between 5.00 and 9.00 PM or at the end of the night-time. Once again, sleep deprivation might have influenced this outcome. It is relevant to report that caffeine consumption after sleep deprivation did not influence fine motor skills that are crucial for vascular anastomosis [22].

The analysis of our data found no difference in terms of overall complications and short-terms outcomes for the pediatric KTs performed during night-time. These results were consistent with the findings in the adult’s population [6, 7]. Even though, the primary outcomes of pediatric KTs performed during days-off to are similar, as already described in the adult population[19], it is relevant to report that the occurrence of post-operative bleedings was higher in KTs performed during days-off. This might be a warning sign of fatigue.

The successful endpoints might be due to a standardization of the surgical procedure and post-operative management together with a well-trained multidisciplinary team, including surgeons, anesthesiologists, nephrologists, and nurses [23].

Moreover, another innovative device could be considered to improve the outcomes of pediatric KTs. The hypothermic machine perfusion of the allografts showed promising results in the adult population [24]. This technology might help the planification of pediatric KTs and, consequently, to reduce the risk of adverse events due to surgeons’ fatigue.

## Conclusions

The number of pediatric KTs performed during after-hours is significant in our experience and is mainly aimed at reducing CIT and to avoid DGF. The incision time during after-hours presented a similar risk of adverse events as compared with ordinary-day interventions. Nevertheless, more caution should be adopted at the end of the ordinary-day shift or in the early morning, especially during days-off because of the fatigue and the sleep deprivation. In these peculiar moments, a consolidated protocol along with a multidisciplinary team of experts could be helpful to avoid hazardous events and to maintain a high standard of success for pediatric KTs.

## Data Availability

The data that support the findings of this study are available from the corresponding author, FG, upon reasonable request.

## Abbreviations

BWR: Body weight ratio
IQR: Inter-quartile range
CIT: Cold ischemia time
KT: Kidney transplantation
DGF: Delayed graft function
PGNF: Primary graft non-function
ESKD: End-stage kidney disease
WIT: Warm ischemia time
